# Artificial Dense Lattices of Magnetic Skyrmions

**DOI:** 10.3390/ma13010099

**Published:** 2019-12-24

**Authors:** Maksim V. Sapozhnikov, Yuri V. Petrov, Nikita S. Gusev, Alexey G. Temiryazev, Olga L. Ermolaeva, Victor L. Mironov, Oleg G. Udalov

**Affiliations:** 1 Institute for physics of microstructures RAS, 603950 Nizhny Novgorod, Russia; msap@ipmras.ru (M.V.S.); gusevns@ipmras.ru (N.S.G.); ermolaeva@ipmras.ru (O.L.E.); mironov@ipmras.ru (V.L.M.); 2 Radio-physic Department, Lobachevsky State University of Nizhny Novgorod, 603950 Nizhny Novgorod, Russia; 3 Physics Department, Saint Petersburg State University, Universitetskaya nab. 7/9, 199034 St. Petersburg, Russia; y.petrov@spbu.ru; 4 Kotel’nikov Institute of Radioengineering and Electronics RAS, Fryazino Branch, 141190 Fryazino, Russia; temiryazev@gmail.com; 5 Physics and Astronomy department, California State University Northridge, Northridge, CA 91330, USA

**Keywords:** magnetic skyrmions, He ion nanomodification, Co/Pt films

## Abstract

Multilayer Co/Pt films with perpendicular magnetic anisotropy are irradiated by focused a He^+^ ion beam to locally reduce the anisotropy value. The irradiated spots with the diameters of 100 and 200 nm are arranged in square lattices with the periods of 200 and 300 nm. The formation of nonuniform magnetic states within the spots was observed by magnetic force microscopy methods. We use the concentric distribution of the irradiation fluence within the spot to obtain the radial modulation of the anisotropy constant. This allows us to induce magnetic skyrmions during magnetization reversal of the system. The skyrmions remained stable at zero external magnetic field at room temperature. Magnetization hysteresis loops of the samples were investigated by magnetooptical methods and the results are in good agreement with micromagnetic simulations.

## 1. Introduction

Soliton-like magnetization distributions (usually referred as “magnetic skyrmions”) in the magnetic materials with the easy-axis anisotropy are known since late 1970s [1,2,3,4,5]. The skyrmions demonstrate high mobility under an applied electric current [6], which can be exploited in spintronic memory and information processing devices. This explains the high degree of interest among researchers into these magnetic textures. The magnetics skyrmions are stabilized by the Dzyaloshinskii–Moriya interaction (DMI) [7,8] in non-centrosymmetric crystals. Since the DMI in these crystals is relatively weak, the skyrmions are stable within a narrow range of temperature and magnetic field only [9]. This hinders the application of such topological objects. Contrarily, ultrathin films of transitional ferromagnetic metal grown on a surface of heavy metal demonstrate strong interfacial DMI stabilizing skyrmions, even at room temperature [10,11]. Therefore, these structures are presently the object of active study.

Magnetic skyrmions can be stabilized in ferromagnetic films, even without the DMI in the case of the proper nanostructuring. One way is to couple the easy axis ferromagnetic film with a nanodot hosting a magnetic vortex [12,13,14,15,16]. In this system the skyrmion is stabilized by an exchange interaction with a magnetic vortex. Another way is to use local modification of the film parameters such as magnetic anisotropy [17], film thickness [18,19], or magnetostatic screening (by using a superconducting [20,21] or paramagnetic [22] capping layer). These methods allow for the initialization of the skyrmions by film magnetization reversal in a uniform external magnetic field. High average topological charge density can be achieved using all these approaches. Note, however, that the skyrmions lose their mobility due to local modification of the film.

The magnetic anisotropy of multilayer Co/Pt films can be modified by He^+^ ion irradiation [23,24]. The irradiation causes a mixing of atoms at the Co/Pt interfaces. This effect decreases the interfacial perpendicular (easy axis) anisotropy. Depending on the irradiation dose, the perpendicular anisotropy can be reduced or even transformed into the easy-plane one. The local irradiation of magnetic film by He [25] or Ga [26] ions allows for the obtaining of a lattice of small magnetic bubbles with different types of domain wall (onion-like, Neel-type and Bloch type). These bubbles may (skyrmions) or may not (skyrmiounium or onion state) have a topological charge. Previously, uniform irradiation within each spot was used. This does not allow for the formation of a regular lattice of skyrmions. Firstly, the topology of magnetization distribution in the uniformly irradiated spots is undefined. Secondly, not all the irradiated spots are switched during the remagnetization process leading to formation of irregular skyrmion ensemble.

In this work, we use the focused ion beam irradiation technique, allowing us to fabricate dense and regular lattices of magnetic skyrmions. We used inhomogeneous concentric irradiation fluence allowing to obtain the anisotropic non-uniform spatial distribution assisting the skyrmions nucleation. The irradiated spots have a ringed outer area with the easy-plane anisotropy, which provokes the formation of the Bloch-type domain wall closed in a ring. This helps to create magnetic bubbles with the topology of a magnetic skyrmion. In the samples with inhomogeneous irradiation, we obtained a dense and highly regular lattice of magnetic skyrmions by magnetization reversal in a uniform magnetic field. Such systems have high topological charge density and are promising for studying of topological transport effects.

## 2. Materials and Methods

We used Co/Pt films consisting of 5 alternating Co (0.5 nm thick) and Pt (1 nm thick) layers for the subsequent preparation of nanostructured samples. The films were deposited by magnetron sputtering in the Ar atmosphere (2 × 10^−3^ Torr) on a glass substrate with 10-nm Pt and 10-nm Ta buffer layer. The layer growth rate was 0.125 and 0.25 nm/s for the Co and Pt layers, respectively. Additional 2-nm thick Pt cap layer was deposited to prevent oxidation.

The magnetization hysteresis loops of the samples were obtained using magneto-optic Kerr effect (MOKE) measurements of the polar geometry in the fields up to 400 mT. A stabilized He-Ne (λ = 632 nm, 5 mW power) laser was used as a light source.

The magnetization distribution of the samples was studied by the magnetic force microscopy (MFM) method. We used a “Solver-Pro” (NT-MDT, Zelenograd, Russia) microscope operating at ambient conditions. We used NSG-11 cantilevers coated with 20 nm of Co layer protected by Ta (10 nm) as the MFM probes. Prior to the measurements, the probes were magnetized along the tip axis in a 1T external magnetic field. The MFM measurements were performed using both the two-pass tapping mode and the noncontact constant-height mode. A phase shift of the cantilever oscillations was registered as an MFM signal. The sample and tip were grounded to avoid an electrostatic interaction [27].

**Figure 1:**
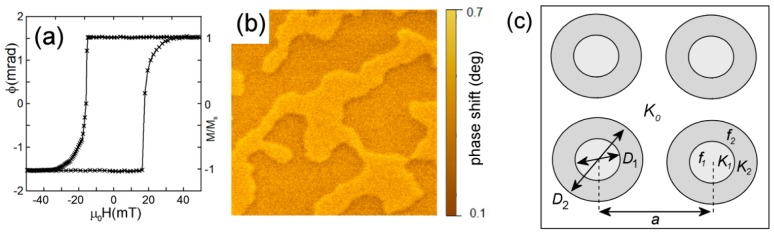
(Color online) (**a**) Typical polar MOKE hysteresis loop of the initial Co/Pt film. It demonstrates easy-axis perpendicular anisotropy. (**b**) 3 × 3 μm^2^ MFM image of labyrinth domain structure in the initial Co/Pt film in a remnant state. (**c**) The geometry of nanomodification: the irradiated spots with the reduced anisotropy form a rectangular lattice with the period of 200 and 300 nm). *D*_1_ and *D*_2_ are the diameters of the regions with different irradiation fluence (*f*_1_ and *f*_2_, correspondingly) and anisotropy (*K*_1_, *K*_2_).

A Carl Zeiss Orion (Carl Zeiss Microscopy, Peabody, MA, USA) helium ion microscope equipped with a Nanomaker pattern generator (Interface Ltd., Moscow, Russia) was used to fabricate rectangular lattices of the irradiated nanospots (below, we refer them as “spots”) with the total area of 150 × 150 μm. The spots have the diameter of 100 and 200 nm with lattice periods of 200 and 300 nm. The geometry of the samples is represented in Figure 1c. The irradiation was performed by a raster method using the focused ion beam having 1 nm size. The scanning step was 0.5 nm. The irradiation fluence varied for different samples from 1 × 10^15^ to 10^16^ ions/cm^2^. The energy of the ions was 30 keV. Two types of the samples were prepared. The first one has the uniform irradiation fluence over the spot. In this sample the ion fluence reduces the anisotropy value within the irradiated area, but the anisotropy remains of the easy-axis type. The second series of samples has concentric distribution of the fluence within each spot. There is the central part irradiated with a small fluence (and therefore having the easy axis anisotropy). There is also the outer ring-shaped part irradiated with high fluence. The anisotropy in this region is of the easy plane type (Figure 1c). We choose the fluences according to [25]. The spot sizes and irradiation fluences for all the samples are summarized in Table 1.

## 3. Experimental Results

Figure 1a shows the typical hysteresis loop of an initial Co/Pt film. The shape of the hysteresis loop is close to rectangular with coercivity of order of 18–20 mT for different samples. In the demagnetized state, the films exhibit a labyrinth domain structure with the domain width of 200–400 nm (Figure 1b). In the remnant state, the films are uniformly magnetized (MFM contrast is absent).

**Figure 2:**
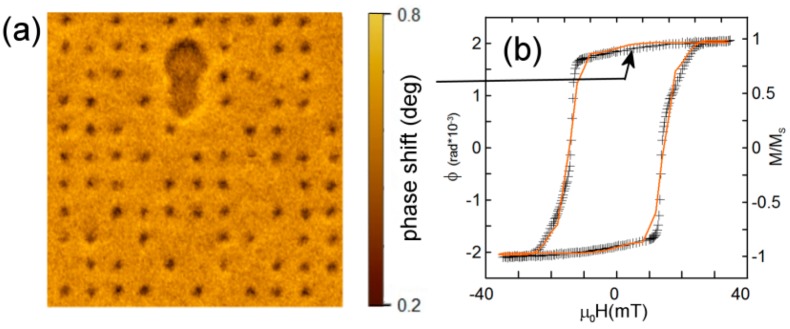
(Color online) (**a**) 2 × 2 μm^2^ MFM image of the remnant magnetic state of the sample irradiated with the uniform He^+^ ion fluence (2.5 × 10^15^ ions/cm^2^) within a spot. (**b**) Magnetization hysteresis loop of the same structure. Crosses are the experimental data. The orange solid line is the loop obtained using numerical simulations.

The MFM image changes drastically after ion irradiation. The typical image of the sample with uniform fluence within the spots (sample No. 1) is represented in Figure 2a. The remnant state (*H* = 0) is shown. Black regions correspond to the irradiated spots. Magnetization direction is switched in these regions, leading to the formation of magnetic bubbles. The following facts should be also mentioned:

- Irregular lattice of bubbles is formed. The bubbles occupy only a part of the irradiated lattice points (Figure 2b). The coercive field of sample No. 1 is ~13 mT, which is less than that of the initial film.
- Reversed domains in non-irradiated part of the film appear even in the remnant state (large black area at the top of the image).
- There are no magnetic bubbles inside the reversed domains. This means that the magnetization within the irradiated spots is oriented in the same direction as the magnetization in the non-irradiated region around. Therefore, we conclude that the irradiated area has the easy-axis perpendicular anisotropy [25].
- The atomic force microscopy (AFM) investigations demonstrate that the flatness of irradiated areas is the same as the flatness of the as-prepared film. So, the irradiation does not change the film topography.
- There is no MFM contrast for the irradiated films in the saturated state. From this, we conclude that magnetization magnitude does not alter due to the irradiation.

**Figure 3:**
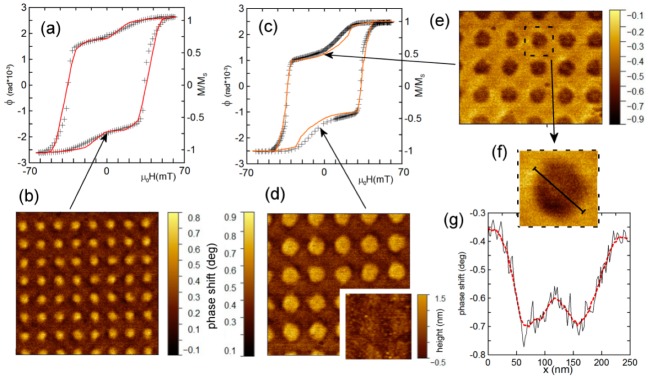
(Color online) (**a**) Hysteresis magnetization loop of the sample with inhomogeneous concentric He^+^ ion fluence (sample No. 2, spot diameters are *D*_1_ = 80 nm, *D*_2_ = 100 nm). Crosses show the experimental data on the polar magneto-optical Kerr effect and the solid red line is the magnetization loop obtained using numerical simulations. (**b**) MFM image of the remnant magnetic state of the sample No. 2. The scan width is 1.6 μm. (**c**) Hysteresis magnetization loop of the sample No. 3 (spot diameters are *D*_1_ = 180 nm, *D*_2_ = 200 nm). Crosses are the experimental MOKE data. The solid orange line is the magnetization loop obtained using numerical simulations. (**d**) MFM image of the remnant magnetic state of the sample No. 3. The scan width is 1.6 μm. Inset represents AFM image with the size of 0.8 μm. (**e**) MFM image of the reversed remnant magnetic state of the sample No. 3. The scan width is 1.6 μm. (**f**) The close view of a single spot. (**g**) The profile of the MFM signal along the line in (**f**). The black curve is the experimental data and the red dashed line is the averaged signal.

It is important to underline that the magnetization reversal does not occurs in all spots. We estimate the reversed spot fraction on the level of 65%. So, the uniform irradiation does not provide a way to realize reliable formation concerning the regular skyrmion lattice. In contrast, the samples with a concentric distribution of the ion fluence (No. 2,3) demonstrate the very regular MFM images (Figure 3b,d). All the spots have reversed magnetization in the remnant state. The hysteresis loop for the samples shown in Figure 3a,c correspond to samples with perpendicular anisotropy. Nevertheless, the hysteresis loops alone do not confirm that there are no small isolated areas with in-plane anisotropy. However, as we mention previously, there is no MFM contrast in the saturated state which evidences that the ion irradiation (with low fluences used in our experiments) does not affect the magnetization magnitude.

A clearly visible step appears close to the remnant state. This step is the consequence of magnetization reversal in the spots. The step is more evident in the case of larger spots (sample No. 3) since they occupy larger area (35% of the whole surface). Magnetization curves for both the samples (2 and 3) demonstrate an increased coercive field, *H*_c_ ~ 30 mT (Figure 3a,c).

The specific feature of the sample with larger irradiated concentric spots (No. 3) is a visible increase of the MFM signal in the central part of the spots (Figure 3f,g).

## 4. Discussion

In order to understand the process of the magnetization reversal and magnetic structure of the remnant state, we carried out micromagnetic simulations utilizing the Object Oriented MicroMagnetic Framework (OOMMF) code [28]. This code is based on a numerical solution of the Landau–Lifshitz–Gilbert (LLG) equations. The simulated system had the geometry corresponding to the experimental samples. A square segment of a magnetic film contains circular spots with reduced anisotropy. The spots are arranged into a square lattice. The segment includes nine unit cells of the lattice. The total size of the system is 600 × 600 nm^2^ for samples No. 1 and 2 and 900 × 900 nm^2^ for sample No. 3. Periodic boundary conditions in the plane of the system are used in order to simulate a large periodic lattice. The thickness of the film is 7.5 nm. Following [29], we consider Co/Pt multilayers as the uniform material with averaged parameters. The step of the numerical grid for samples 1, 2 and 3 is 600/512 = 1.17 nm and 900/512 = 1.76 nm, correspondingly. It is smaller than the characteristic thickness of the domain wall in the system. We assume uniform magnetization distribution across the film thickness. The saturation magnetization *M*_s_ and the exchange coupling constant *A* are 2 × 10^5^ A/m and 2.5 × 10^−13^ J/m, respectively. They were chosen according to our previous work [25], where good correlation between experimental data and simulations was demonstrated. The anisotropy constants were chosen to get the best fit of our experimental data. The average values of the anisotropy used in the simulations are presented in Table 2. In real samples the anisotropy varies of across the film. To take this into account, we carried out several numerical simulations of each system with slightly different anisotropy values (*K*_0_—anisotropy of non-irradiated film, *K*_1_ and *K*_2_ anisotropies of irradiated areas) and averaged the obtained magnetization curves. In each simulation, we add a small anisotropy (*K*_r_ = *K*_0_ × 0.01) randomly distributed over the film to introduce an inhomogeneity initializing magnetization reversal.

We calculated the magnetization of all the samples as a function of the external magnetic field perpendicular to the film. The simulated curves are shown in Figure 2b and Figure 3a,b by solid orange lines. They demonstrate good agreement with the experimental data.

**Figure 4:**
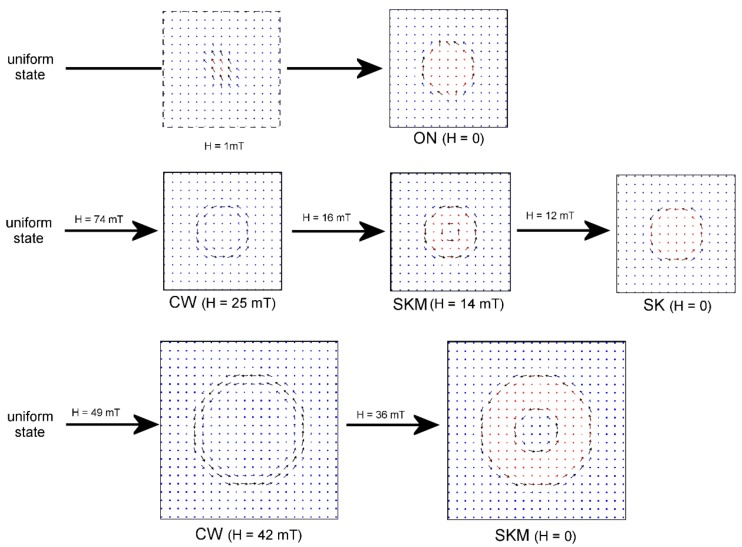
(Color online) The schematic representations of the magnetization state transformations in the unit cell of the spots lattice during the magnetization reversal (results of numerical simulations). The upper line is for the system with uniform anisotropy within a spot (sample 1, *D*_1_ = 100 nm, a = 200 nm). ON stands for onion state. The middle line is for the sample with concentric distribution of the anisotropy (sample 2, *D*_2_ = 100 nm, a = 200 nm). The bottom line corresponds to the sample 3 (*D*_1_ = 200 nm, a = 300 nm). CW means circular wall, SKM stands for skyrmionium, and SK is for skyrmion. The field values shown above the arrow correspond to the transition between different states. The field at which a particular magnetization distribution is obtained is shown below the corresponding distribution. The image surrounded by a dotted line does not show a stable state but an instant snapshot of the magnetization turnover in the uniformly irradiated spot.

How does the magnetization reversal process take place? The magnetization switching process in sample No 1 is illustrated in the upper panel in Figure 4. The shown simulated magnetization distributions are obtained using the anisotropy constants *K*_0_ = 3.2 × 10^4^ J/m^3^, *K*_1_ = 2.4 × 10^4^ J/m^3^. Simulations start at a high positive magnetic field and uniform magnetization distribution. Decreasing the external field leads to the turnover of the magnetization in the spot with the reduced anisotropy. Eventually, the domain wall with two Bloch lines is formed. Such a domain wall corresponds to the so-called “onion state”. The final remnant (zero external field) magnetization distribution is a trivial (topologically uncharged) state [18]. The film still has the perpendicular anisotropy within the spot. Nevertheless, the demagnetizing fields lead to formation of the onion states even before the external magnetic field reaches zero. In simulations, we see that some spots do not switch or switch into a skyrmion state. This happens due to the fluctuations of the anisotropy and the magnetostatic interaction between different spots. So, an irregular lattice of onion states, skyrmions, and unswitched spots is realized. This is in agreement with our experimental observations.

The Bloch lines in the domain wall of the bubble facilitate the formation of a labyrinth domain structure (at around −13 mT) followed by complete magnetization reversal at negative fields in the order of −18 mT, which is even less than the coercive field of the initial film. In the experiment we observe the same decrease of the coercivity (compare Figure 1a and Figure 2b).

The situation becomes quite different in the samples with concentric irradiation fluences (Figure 4, central row). In this case, the outer ring of a spot has an easy plane anisotropy, while the central part of the spot has an easy-axis one. Therefore, the process of the magnetization reversal begins with the formation of the circular domain in outer ring (Figure 4, CW). The consequent decrease of the field leads to the tilting of the magnetization in the ring resulting in magnetization reversal inside the spot. The circular domain usually referred as “skyrmionium” is finally formed (Figure 4, SKM) [30,31]. Actually, the “skyrmionium” is a topologically trivial state, which can be considered as a pair of concentric magnetic skyrmions with opposite topological charges. Since the magnetization reversal starts in the outer region but not in the central one, the resulting domain wall does not have Bloch-line defects. At a critical external field *H*_c_ the central part of the skyrmionium switches and turns into a skyrmion (Figure 4, SK). *H*_c_ depends on the anisotropy values but it is always positive for the anisotropy constants used in our simulations (see Table 2). So, at zero field one has a dense lattice of magnetic skyrmions. The absolute value of the field needed for the formation of the labyrinth domain structure increases up to 30 mT (the field is negative). The complete magnetization reversal occurs at −50 mT in this case. Note that the fluctuations of anisotropy in the system smears the transitions between different magnetic states. Therefore, the field magnitude indicated above is the approximate one.

In the spots with a larger size, the skyrmionium state has more space for its central part. Therefore, the increase of the irradiated region diameter enhances the stability of skyrmioniums. In the case of the wide (*D* = 200 nm) spots, the skyrmionium remains stable even at zero field. The simulations demonstrate SKM → SK transition in negative fields, so the remnant state is the lattice of skyrmioniums. In the skyrmionium state, in large spots, the central part is not switched while the outer part is switched (see the SKM state in Figure 4). This induces an increase of the MFM signal seen in experiment (Figure 3f,g).

We should also notice that the interfacial DMI is possible in the Co/Pt multilayers [32]. However, the observation of the labyrinth domain structure in the non-irradiated film evidences that the DMI in the as-prepared film is absent or too small to stabilize skyrmions.

## 5. Conclusions

We have studied the magnetization reversal and apparent magnetic states in multilayer Co/Pt films nanopatterned by a He^+^ focused ion beam. Films with dense lattice of irradiated nanospots were fabricated. Two types of films were prepared with homogeneous and inhomogeneous irradiation of a single spot. In the case of the uniform fluence in the irradiated spot, the apparent magnetic bubbles do not have the topology of a skyrmion. Contrarily, in the case of the concentric distribution of irradiation, fluence favors the formation of the dense lattice of magnetic skyrmioniums or skyrmions. In the large irradiated spots, the skyrmioniums are stable, while in the small spots, skyrmions appear. The density of a skyrmions topological charge in the artificial lattice is 25 μm^−2^, which is comparable with the charge density in natural chiral magnetic materials.

## Figures and Tables

**Table 1:** Parameters of the investigated nanostructured films: *D*_1_ and *f*_1_ are the diameter and the irradiation fluence of the central part of a spot (Figure 1), *D*_2_ and *f*_2_ are the same for the outer ringed region, and *a* is the lattice period.

No	*D*_1_ (nm)	*f*_1_ (cm^−2^)	*D*_2_ (nm)	*f*_2_ (cm^−2^)	*a* (nm)
1	100	2 × 10^15^	-	-	200
2	80	10^15^	100	10^16^	200
3	180	2 × 10^15^	200	4 × 10^15^	300

**Table 2:** Anisotropy constants used in numerical simulations of the nanostructured films.

No	*K*_0_ (J/m^3^ × 10^4^)	*K*_1_ (J/m^3^ × 10^4^)	*K*_2_ (J/m^3^ × 10^4^)
1	3.2 ± 0.2	2.4 ± 0.2	-
2	3.2 ± 0.2	2.7 ± 0.2	1.0
3	3.2 ± 0.2	2.4 ± 0.2	1.4
